# Intussusception
1Read at the Meeting of the Bristol Medico-Chirurgical Society on December 13th, 1893. A Report of the Discussion Followed is Given under "Meetings of Societies."


**Published:** 1894-03

**Authors:** Arthur W. Prichard

**Affiliations:** Senior Surgeon to the Bristol Royal Infirmary; Lecturer on Practical Surgery in University College, Bristol


THE BRISTOL

### Medico-Chirurgical Journal.

MARCH, 1894.

### INTUSSUSCEPTION.[1]

BY

ARTHUR W. PRICHARD, M.R.C.S. Eng.,

*Senior Surgeon to the Bristol Royal Infirmary; Lecturer on Practical Surgery in University College, Bristol.*

My duty to-night is to introduce the subject of Intussusception, and to suggest ideas that subsequent speakers will sustain and enlarge upon or refute. I do not at all profess that any, or at all events many, of my suggestions are original; but by recalling attention to the details, I hope to elicit the opinions of those who have given the subject the consideration it deserves.

The plan of my paper will be this:

1st, to say a few words as to the condition and probable causes;

2nd, to recount three cases;

3rd, very briefly to run over the symptoms necessary for diagnosis;

4th, to discuss the treatment.

First, then, as to the condition and probable causes—and I must again ask you to pardon me if I say many things familiar

[1] Read at the meeting of the Bristol Medico-Chirurgical Society on December 13th, 1893. A report of the discussion which followed is given under "Meetings of Societies."

to you all. By intussusception we mean the condition in which a part of the bowel with its mesentery slips into and is, so to speak, swallowed by the part of the bowel immediately below it. This may occur at any point in the intestinal tract, but is most common in the neighbourhood of the ileo-cæcal valve. In the case where the small intestine alone is involved, the invagination is usually short, and this—the enteric variety—occurs in 30 per cent. of the whole cases. The colic variety—or that in which the large bowel alone is concerned—occurs in 18 per cent., and naturally we find it most common in the descending colon and sigmoid flexure. The ileo-cæcal variety is the commonest, forming 44 per cent., and is that in which the ileum enters the cæcum and then advances into the ileo-cæcal valve at its head, the ascending colon doubling inside itself. This may be extensive, and in rare cases may occupy the whole length of the large bowel. Another variety is that called ileo-colic, and is where the cæcum caput coli remains *in situ*, and the small intestine passes through the valve into the larger. This occurs in about 8 per cent. of the recorded cases.

With regard to the cause: to me it seems wonderful, when we think how active and apparently partial and irregular peristalsis is, that intussusception does not more commonly occur. Anything that interferes with the proper regulation of the rhythmical movement of the intestine may cause it, and increased excitement in one part from the presence of an irritating substance—such as a piece of undigested food—probably commonly gives rise to it. Occasionally a polypus or other tumour of the intestinal wall has been shown to have excited it. Decreased or disordered intestinal movements from want of proper nerve-control, such as may occur in certain cerebral lesions, may predispose to it; and under this head we must place that curious phenomenon of intussusception of the dying, which is not uncommon. There is on the table a very good specimen of this condition, showing multiple intussuscepta, easily reducible, and not all pointing in the same direction, in some places the upper segment of the bowel having slipped inside the lower, and in others the lower inside the upper. But of all causes, I think that, in children at all events, it is very likely that the condition is more or less directly due to external violence. We know that a great proportion of the cases occur in children. It is very common under the age of one year, and more than half the cases occur in those under the age of 10. When we think of the way in which infants are hurriedly snatched up, and carried doubled up with the hand or arm round the abdomen, can we be astonished at a part of the intestine becoming injured and perhaps paralysed by the undue pressure through the thin abdominal wall? Irregular peristalsis thus set up may be the cause of an intussusception, which manifests itself by the child screaming with abdominal pain an hour or two afterwards, the hasty lift being forgotten from constant usage, or perhaps, on the part of the nursemaid, denied. I cannot help saying in this place that I think it quite possible that intussusception occurs more frequently than we generally suppose, because of the very ready way in which it may be caused. A great many children suffer from severe abdominal pain that may or may not be followed by vomiting and diarrhœa, symptoms which give way to an opiate. Is it not likely that some of these cases are due to a small intussusception, which rights itself and gives rise to no subsequent trouble? When the pain is referred definitely to the region of the ileo-cæcal valve, the attack being paroxysmal and severe, it is sometimes recognised as being an instance of ileo-cæcal intussusception, and a tumour that disappears spontaneously can be felt in that neighbourhood. I am, however, inclined to think that we might find more cases of intussusception if we had opportunities of palpating the abdomen before Nature cured the patient.

I will now relate two cases that have been under my care at the Infirmary—the first a few years ago, and the second quite recently:

A child under the age of two was admitted into Ward 20 with severe abdominal symptoms. I am sorry I cannot give exact details of the case, as the record of it has been unfortunately mislaid. On examination of the abdomen a resisting mass was felt about the umbilicus and in the left flank. With the finger in the rectum, I could feel that a soft tumour filled the gut, and on pulling this a little downwards I could demonstrate that the apex of the tumour just inside the anus was the ileo-cæcal valve. Under chloroform, I was able to push this easily up into the abdomen with my finger, and then with the help of a small pair of bellows, the nozzle of which was passed into the rectum and the anus held closely all round by a towel to prevent the escape of air, by inflation and manipulation I succeeded in nearly, if not quite, restoring the parts to their normal position. I could follow the tumour, and found it almost disappear somewhere near the liver. It was not a permanent cure, however, for in a few days the process had to be repeated; after that the patient was taken out, so that I cannot give the result of the case.

The second case was that of John E., aged about 30, admitted in the morning of October 14th last. He said he had been ailing for three days. He had been sick and had not passed anything from the bowels. He had had an inguinal hernia of the left side, but when I saw him, at one o'clock in the day, there was no sign of it. He was a rather fat man, with very slight distension of the abdomen, vomiting frequently—the vomit at first being not distinctly stercoraceous, but it soon became so. He had passed neither motion nor flatus for three days. There was nothing in the rectum, and nothing very definite could be felt on palpating the abdomen. There was no great pain. The patient was in such a condition, with frequent and feeble pulse and blue and cold extremities, that it was deemed inadvisable to operate at once; and thinking he might be suffering greatly from the shock caused by a journey, I directed that he should be kept warm in bed for a few hours, and should have stimulating and nutrient enemata. These he did not retain, but none of the contents of the intestine came with them. At five o'clock he had not improved, and after consultation, and at the patient's wish, I thought it best to give him the remote chance of life by operation rather than let him die from acute intestinal obstruction unrelieved. Under chloroform, I opened the abdomen in the middle line just below the umbilicus, and my fingers at once came upon a piece of distended and hard gut. This was lying immediately under the incision, and there was no difficulty in lifting it out of the abdomen. It was very dark-red, about six inches long, and to the touch solid. It was at once evident that the case was one of intussusception of the small intestine, and I was astonished at the thickness of the twisted mesentery that led into the tumour with the entering gut; it was a tight band of firm tissue as thick as one's forefinger. I made several attempts to pull it out, and though I was able to move by manipulation through the outside wall the apex of the invaginated part, I could not dislodge the gut, which seemed to be held and strangulated just inside the entrance. I therefore put enterectomy clamps on the healthy part of the intestine above and below the tumour and cut it through, and was proceeding to secure the mesentery when the patient showed signs that further interference could be of no avail, and a few minutes later he died on the table. This was about twenty minutes from the commencement of anæsthesia. The specimen in its recent state showed how great had been the effort Nature had made to dislodge the obstruction. The intussusceptum was quite black, with a layer of coagulated blood outside it. It was, as we found next day, about five feet from the pylorus.

Another case to which I may refer is a remarkable one of my father's. It is fully published, with an illustration, in the *British Medical Journal* of April 13th, 1858, and is an instance of intussusception in which the patient, a child of five, passed a sphacelated part of the bowel *per anum*, and lived for five months after. He died from starvation on account of a large fistulous opening that remained between the duodenum and the descending colon, the result evidently of general peritonitis that followed upon the casting off of the strangulated intussusceptum. This case was also notable because at one period of his illness the boy constantly passed fæcal matter and gas *per urethram*, but the entero-vesical communication had closed before his death.

I have references to three or four other cases, but as these were under the care of my colleagues at the Infirmary, I hope they will speak about them.

A few words as to the most prominent symptoms will not be out of place, for in cases of serious abdominal trouble it is very possible to make mistakes, and it requires considerable experience and close attention to details to be able to describe beforehand with anything like exactitude the condition that will be found after opening the abdomen. The points that will help us most in the diagnosis of intussusception are these: that it is more common in males, and occurs chiefly in the young. The great majority of cases that come under the category of intestinal obstruction in children under three are instances of acute intussusception. Chronic intussusception occurs more often in males during adult life. The chief symptom is pain. It comes suddenly and is very severe—intermittent at first, and then localised round the tumour. There is no regular premonitory symptom. There may be diarrhœa. This diarrhœa usually continues, and in most cases is accompanied by a bloody discharge. Complete obstruction is extremely rare. Vomiting appears early, and is not, as a rule, copious. Tenesmus is generally very severe. Collapse is early, and the abdomen is flaccid unless peritonitis has set in. The most definite sign is a tumour, described usually as "sausage-shaped." This, felt through the flaccid abdominal wall, is pathognomonic of the condition. It usually occupies the line of the colon. It is dull on percussion and movable.

Such are the chief symptoms, as gathered from books, by which we can recognise a case of intussusception, and I shall hope to hear this evening some remarks on their reliability. In the second case I related, I can only come to the conclusion that it was an exception to the rule in almost every particular, as, except early collapse and vomiting, most of the generally recognised signs and symptoms were absent or masked.

Lastly, as regards treatment: The time that has elapsed since the invagination took place is one of the chief points to consider in determining what course of treatment had best be pursued. It is difficult, and would probably do harm, to promulgate a rule on the subject; but I believe that most surgeons will agree that in acute cases about 48 hours is the limit of time during which we can hope to effect a cure by manipulation and injections, or both combined. After this period probably adhesions have formed between the serous coats, and these adhesions are strong enough to prevent the release of the intussusception without grave danger to the bowel below from the necessary force of the injection. If the case is left to Nature, or to Nature assisted by belladonna or opium and a suitable diet, it is possible for the invaginated part to slough off and be passed *per anum*, and the patient escape with his life. This does not occur often, and never in very young infants: it has only been recorded in children, above the age of one year, in whom there has been sufficient vitality to overcome the shock of the accident. In chronic cases there is a difference. In these probably the intussusception shifts and slips partly out, and adhesions are prevented from forming and the intussusception does not become strangulated. In them reduction may be repeatedly made, and as, in cases of prolapse of the rectum, after several trials reduction on some one occasion is permanently successful. In the treatment of these chronic cases I think there is a chance for the exhibition of other than surgical skill, at all events as regards the prevention by diet and drugs of a recurrence of the catastrophe.

In the operation of reduction by pressure from the lower end of the bowel, air or water or a solid may be tried. The last would only be useful where the intussusception can be felt with the finger in the rectum. An oiled, padded bougie pressed up against the obstruction has been successful; but higher than the lower part of the sigmoid flexure this is hardly likely to succeed. Most commonly air or water is employed, sometimes oil or milk. In a great many cases injection of either has effected a reduction, and there is a great difference of opinion as to which should be tried. The patient is of course fully anæsthetised, and means are taken to prevent escape from the anus. My opinion is that one of the dangers lies in these means being too perfect. We ought, I think, to be able to estimate the degree of pressure on the walls of the bowel, so as to prevent this being excessive. Injection of air by a Higginson's syringe or a small pair of bellows could probably not burst the bowel, and yet will give sufficient force for the purpose. By this means, as we have proved on the cadaver, the large intestine can be easily filled, and the air passes readily through the ileo-cæcal valve. Water, milk, or oil can be injected by a syringe or by an irrigator; the latter is the better instrument to choose, as we can regulate the pressure by the height of the irrigator. If the level of the water in the irrigator is three feet above the level of the intestine, the pressure on the square inch of intestine is about $1\frac{1}{2}$ lbs.; and by experiments which Mr. Mole has kindly made in connection with this paper, it was proved that the ileo-cæcal valve formed no barrier to the passage of water into the small intestine. Mr. Mole will, I hope, give us the results of his experiments, which are remarkable, and which, in my opinion, show that there is grave danger of rupture from the employment of too much pressure. I think a child would stand a far better chance of getting well after an abdominal section and the disentangling of the invagination by the fingers, than by the influx of water, if the irrigator had to be held higher than three feet to bring about the reduction; and yet in a well-known book on children's diseases it is directed that the irrigator should be held high above the bed.

Where these means have failed or it has been decided not to try them, the choice lies between abdominal section and leaving the patient to the chance of a spontaneous cure by the sloughing off of the intussuscepted portion. We should probably all choose the former. The abdomen should be opened in the middle line below the umbilicus, and the offending portion of the intestine, which can usually be easily reached, brought to the surface. Gentle, firm traction on the intussusception, combined with taxis on the blunt lower end of the tumour, in many cases has resulted in the parts assuming their normal relation. Should it not be possible to pull the invaginated part out without applying undue force, four courses are open to us; and as the patient is probably in a condition where prolongation of any operation would greatly lessen his chance of life, we should probably choose the procedure that can be most quickly accomplished. In the first place, we can make an artificial anus with resection of the whole tumour, by sewing the healthy part of the intestine just above and below the tumour to the abdominal wall and cutting off the mass, taking care to secure the vessels of the mesentery. This operation has been done, but I do not know if ever successfully. Should the patient survive, the artificial anus could be subsequently operated on. Secondly, we could resect the tumour and unite the cut ends of the intestine and replace it in the abdominal cavity; a proceeding that it would take some time to accomplish. Thirdly, where adhesions have formed, we could strengthen those adhesions by stitching the turned-in edge of the intussuscipiens to the contiguous surface of the intussusceptum, and then either leave the intussusceptum to its fate or open the intussuscipiens by a longitudinal incision along its free border, and divide and remove the strangulated bowel within, and then sew up the incision. This would probably be only successful where a small piece of intestine has become displaced. Fourthly, where a large invagination has taken place and is strangulated, the only possible chance is to make an artificial anus in the healthy part of the gut above.

The results of the cases that have been published of enterectomy for intussusception are far from encouraging; but it is not a common condition, and as reasonable methods of operating in such cases are suggested and tried, and as apparatus is perfected, we may, I think, hope that some such improvement will manifest itself in them as has of late years appeared in the operative treatment of many serious abdominal lesions; and I hope, in conclusion, that the discussion to-night will not only be of scientific interest, but may give us some good reasons for deciding what course of treatment we had best adopt in future for the greatest welfare of our patients.

---

